# Factors Associated With Severe Gastrointestinal Diagnoses in Children With SARS-CoV-2 Infection or Multisystem Inflammatory Syndrome

**DOI:** 10.1001/jamanetworkopen.2021.39974

**Published:** 2021-12-20

**Authors:** Andrea Lo Vecchio, Silvia Garazzino, Andrea Smarrazzo, Elisabetta Venturini, Marco Poeta, Paola Berlese, Marco Denina, Antonella Meini, Samantha Bosis, Luisa Galli, Salvatore Cazzato, Giangiacomo Nicolini, Gianluca Vergine, Roberta Giacchero, Giuseppina Ballardini, Icilio Dodi, Filippo Maria Salvini, Paolo Manzoni, Giuliana Ferrante, Vera Quadri, Andrea Campana, Raffaele Badolato, Alberto Villani, Alfredo Guarino, Guido Castelli Gattinara

**Affiliations:** 1 Department of Translational Medical Sciences, Section of Paediatrics, University of Naples Federico II, Naples, Italy; 2 Paediatric Infectious Diseases Unit, Regina Margherita Children’s Hospital, University of Turin, Turin, Italy; 3 Ospedale Bambino Gesù IRCCS, Rome, Italy; 4 Infectious Diseases Unit, Meyer Children’s University Hospital, Florence, Italy; 5 Department of Paediatrics, Cà Foncello Hospital, Treviso, Italy; 6 Department of Experimental and Clinical Sciences, Paediatric Clinic, University of Brescia, Brescia, Italy; 7 Fondazione IRCCS Cà Granda Ospedale Maggiore Policlinico, Milan, Italy; 8 Department of Health Sciences, University of Florence, Florence, Italy; 9 Paediatric Unit, Department of Mother and Child Health, Salesi Children's Hospital, Ancona, Italy; 10 Unità Operativa Complessa Pediatria, San Martino Hospital, Belluno, Italy; 11 Unità Operativa Complessa Pediatria, Ospedale degli Infermi di Rimini, Rimini, Italy; 12 Unità Operativa Complessa Pediatria, Azienda Sanitaria Territoriale di Lodi, Lodi, Italy; 13 Unità Operativa Complessa Pediatria, Ospedale Castelli, Verbania, Italy; 14 Emergency and General Paediatric Unit, Pietro Barilla Children’s Hospital, Parma, Italy; 15 Paediatrics Division, Azienda Sanitaria Territoriale Grande Ospedale Metropolitano Niguarda, Milan, Italy; 16 Division of Paediatrics and Neonatology, Department of Maternal, Neonatal, and Infant Health, Ospedale degli Infermi, Azienda Sanitaria Locale Biella, Ponderano, Biella, Italy; 17 Department of Maternal and Child Health, University of Palermo, Palermo, Italy; 18 Azienda Sanitaria Territoriale Papa Giovanni XXIII, Bergamo, Italy

## Abstract

**Importance:**

Severe gastrointestinal (GI) manifestations have been sporadically reported in children with COVID-19; however, their frequency and clinical outcome are unknown.

**Objective:**

To describe the clinical, radiological, and histopathologic characteristics of children with COVID-19 presenting with severe GI manifestations to identify factors associated with a severe outcome.

**Design, Setting, and Participants:**

A multicenter retrospective cohort study (February 25, 2020, to January 20, 2021) enrolled inpatient and outpatient children (aged <18 years) with acute SARS-CoV-2 infection, confirmed by positive real-time reverse-transcriptase–polymerase chain reaction on nasopharyngeal swab or fulfilling the US Centers for Disease Control and Prevention criteria for multisystem inflammatory syndrome in children (MIS-C). The study was conducted by pediatricians working in primary care or hospitals in Italy participating in the COVID-19 Registry of the Italian Society of Pediatric Infectious Diseases.

**Main Outcomes and Measures:**

The occurrence of severe GI manifestations, defined by a medical and/or radiological diagnosis of acute abdomen, appendicitis (complicated or not by perforation and/or peritonitis), intussusception, pancreatitis, abdominal fluid collection, and diffuse adenomesenteritis requiring surgical consultation, occurring during or within 4 to 6 weeks after infection with SARS-CoV-2 infection. Logistic regression was used to estimate odds ratios (ORs) with 95% CIs of factors potentially associated with severe outcomes.

**Results:**

Overall, 685 children (386 boys [56.4%]; median age, 7.3 [IQR, 1.6-12.4] years) were included. Of these children, 628 (91.7%) were diagnosed with acute SARS-CoV-2 infection and 57 (8.3%) with MIS-C. The presence of GI symptoms was associated with a higher chance of hospitalization (OR, 2.64; 95% CI, 1.89-3.69) and intensive care unit admission (OR, 3.90; 95% CI, 1.98–7.68). Overall, 65 children (9.5%) showed severe GI involvement, including disseminated adenomesenteritis (39.6%), appendicitis (33.5%), abdominal fluid collection (21.3%), pancreatitis (6.9%), or intussusception (4.6%). Twenty-seven of these 65 children (41.5%) underwent surgery. Severe GI manifestations were associated with the child’s age (5-10 years: OR, 8.33; 95% CI, 2.62-26.5; >10 years: OR, 6.37; 95% CI, 2.12-19.1, compared with preschool-age), abdominal pain (adjusted OR [aOR], 34.5; 95% CI, 10.1-118), lymphopenia (aOR, 8.93; 95% CI, 3.03-26.3), or MIS-C (aOR, 6.28; 95% CI, 1.92-20.5). Diarrhea was associated with a higher chance of adenomesenteritis (aOR, 3.13; 95% CI, 1.08-9.12) or abdominal fluid collection (aOR, 3.22; 95% CI, 1.03-10.0).

**Conclusions and Relevance:**

In this multicenter cohort study of Italian children with SARS-CoV-2 infection or MIS-C, 9.5% of the children had severe GI involvement, frequently associated with MIS-C. These findings suggest that prompt identification may improve the management of serious complications.

## Key Points

**Question:**

Is COVID-19 associated with severe gastrointestinal manifestations in children?

**Findings:**

In this multicenter cohort study of 685 Italian children with COVID-19, 10% showed severe gastrointestinal involvement characterized by diffuse adeno-mesenteritis, appendicitis, abdominal fluid collection, ileal intussusception, or pancreatitis. Children older than 5 years and those presenting with abdominal pain, leukopenia, or receiving a diagnosis of multisystem inflammatory syndrome were more likely to have severe gastrointestinal manifestations.

**Meaning:**

Severe gastrointestinal involvement is not uncommon in children with COVID-19, and awareness about its frequency and presentation may help practitioners to appropriately manage children at risk of severe outcomes.

## Teaser

This cohort study assesses the clinical, radiological, and histopathologic gastrointestinal characteristics of a large cohort of children with acute SARS-CoV-2 infection or multisystem inflammatory syndrome in children to identify factors associated with severe gastrointestinal manifestations and describes their clinical course and outcome.

## Introduction

The gastrointestinal (GI) tract is one of the target organs affected by SARS-CoV-2. The colocalization of angiotensin-converting enzyme 2 and the proteaselike transmembrane serine protease 2, essential receptors for SARS-CoV-2 cell binding and internalization, has been noted in the human GI tract.^1,2^ The presence of isolated GI symptoms in some patients with SARS-CoV-2 infection, as well as the prolonged fecal shedding reported in neonates and children, supports the hypothesis of a fecal-oral transmission of SARS-CoV-2.^3^

The incidence of GI symptoms in patients with SARS-CoV-2 infection varies according to age, underlying conditions, and setting. Compared with adults, children are more likely to present with GI symptoms.^4^ About a quarter of children with acute SARS-CoV-2 infection and almost 90% receiving a diagnosis of multisystem inflammatory syndrome in children (MIS-C) develop diarrhea, nausea, vomiting, or abdominal pain.^5,6^ Evidence from a large multicenter study in Italy showed that diarrhea occurs in about 15% of children with COVID-19, with a more frequent presentation in infancy, and vomiting (10%) or abdominal pain (8%) have a higher frequency in school-aged children.^7^

The presence of GI symptoms at disease onset has been related to a higher probability of a severe clinical course, intensive care unit admission, and mortality.^8,9^ Severe GI involvement characterized by appendicitislike presentation, diffuse mesenterial inflammation, or terminal ileitis has been sporadically reported during the first and second pandemic waves.^10,11,12^ However, the evidence currently available does not allow estimation of the frequency and clinical course of severe GI presentation in children with COVID-19.

A better knowledge of the factors associated with severe GI manifestations, as well as an increased awareness of their clinical course and outcome, may provide supporting information to practitioners working either in the setting of the emergency department or in primary care. This information may be of certain relevance now that the prevalence of COVID-19 in the pediatric population is increasing^13^ and the vaccination coverage is still far from that reported in adults.

In that scenario, we investigated the clinical, radiological, and histopathologic GI characteristics of a large cohort of children with acute SARS-CoV-2 infection or MIS-C, with the aim of identifying factors associated with severe GI manifestations and describing their clinical course and outcome.

## Methods

### Study Protocol and Case Definition

The present retrospective cohort study is part of a larger initiative promoted by the Italian Society of Pediatric Infectious Diseases aimed at investigating the epidemiological, clinical, and therapeutic aspects of SARS-CoV-2 infection in children and adolescents.^9,14^ We thereby focused on GI manifestations in this population with a diagnosis of acute symptomatic SARS-CoV-2 infection or MIS-C. This study was undertaken in accordance with Good Clinical Practice guidelines and the Declaration of Helsinki.^15^ The study protocol was approved by the ethical committee of the University of Naples Federico II and by independent ethics committees and/or institutional review boards of each enrolling center. Patients were included after providing their written informed consent, when appropriate, or consent from their parents/caregivers. This study followed the Strengthening the Reporting of Observational Studies in Epidemiology (STROBE) reporting guideline for cohort studies.

The diagnosis of infection was established in the presence of suggestive symptoms and at least 1 respiratory specimen positive for SARS-CoV-2 nucleic acid using a validated real-time reverse-transcriptase–polymerase chain reaction assay. Few children with highly suggestive symptoms and from homes with COVID-19–positive occupants were diagnosed through detection of immunoglobulin M and immunoglobulin G against SARS-CoV-2 during the first wave of the pandemic. Multisystem inflammatory syndrome was defined according to US Centers for Disease Control and Prevention criteria.^16^

### Population, Data Collection, and Clinical Outcomes

We retrospectively analyzed data from a large cohort of children younger than 18 years who received a diagnosis of COVID-19 between February 25, 2020, and January 20, 2021, in 54 Italian institutions as well as by primary care pediatricians (eFigure 1 in Supplement 1). A single researcher for each institution collected and deidentified clinical data, and all data were merged into a single electronic database specifically designed for statistical analysis. For the study purpose, only data on children with symptoms were included. In addition to the clinical data, demographic characteristics (age, sex, and race and ethnicity) were obtained.

All patients in the data set were considered for inclusion in the study and revised for the presence of GI symptoms, including diarrhea, vomiting, nausea and anorexia, abdominal pain and distention, or suspected surgical conditions. The occurrence of severe GI symptoms and diagnoses was considered the primary outcome of the present study. Children were excluded from the analysis if inaccuracy in reporting clinical data did not allow patient classification and appropriate definition of the primary outcome (eFigure 1 in Supplement 1). An adequate follow-up period to outline the clinical course and outcome of the infection was set to 2 weeks or longer.

To identify possible risk factors associated with severe GI manifestations, all children were classified into 3 subgroups: (1) symptomatic without GI symptoms, (2) mild to moderate GI symptoms, and (3) severe GI clinical manifestations. The latter group included all children who received a medical and/or radiological diagnosis of acute abdomen, appendicitis, ileal intussusception, pancreatitis, abdominal fluid collections, and diffuse adenomesenteritis requiring surgical consultation that was temporally associated with SARS-CoV-2 infection.

Additional information was collected for patients with severe GI manifestations, including medical reports of abdominal ultrasonography, computed tomography, surgical intervention, intra-abdominal fluid/tissue samples collected at the time of surgery, and histologic test findings. Two researchers (A.L.V. and M.P.) independently reviewed the original reports and the classification of patients according to the severity of GI presentation; in addition, a pathologist unaware of patients’ diagnosis and clinical condition reviewed histologic test findings in case of disagreement. Further details about the overall methods have been published.^9,14^

### Statistical Analysis

Continuous variables are reported as mean (SD) or median (IQR) according to their distribution and compared using *t* test or Mann-Whitney test, as appropriate. Categorical variables expressed as frequencies and percentages were compared using Fisher exact test or χ^2^ test. Univariable and multivariable logistic regression analyses were used to identify the variables associated with a severe GI outcome, and risk is expressed as crude odds ratio (OR) and adjusted OR (aOR) with 95% CI. Multivariable analysis included age, sex, GI symptoms, MIS-C, and variables found to have a significance level of *P* ≤ .10 in the univariate analysis. Because MIS-C was included in the multivariable analysis, single parameters necessary for the definition of this syndrome (ie, elevated leukocyte, C-reactive protein, or ferritin levels) were not included to avoid biases. The primary analysis was undertaken under the principle of complete case analysis. A best-worst case approach was used as a sensitivity analysis in case of missing data exceeding 10% of cases. Two-sided *P* values <.05 were considered significant. Statistical analysis was performed using SPSS Statistics for Windows, version 25.0 (IBM Corp).

## Results

### Study Population

Overall, 685 children (386 boys [56.4%]; 299 girls [43.6%]; median age, 7.3 [IQR 1.6-12.4] years) were included in the present study. Six-hundred twenty-eight children (91.7%) had acute SARS-CoV-2 infection and 57 (8.3%) received a diagnosis of MIS-C. General characteristics of the study population are reported in Table 1. A total of 9 Black, 2 Hispanic, 1 Asian Indian, and 673 White children were included.

**Table 1.** Characteristics of the Study Population According to Severity of GI Symptoms

General characteristic	No. (%)				*P* value^a^
	Total (N = 685)	Children without GI involvement (n = 428 [62.5%])	GI clinical manifestations		
			Mild to moderate (n = 192 [28.0%])	Severe (n = 65 [9.5%])	
Age, median (IQR), y	7.3 (1.6-12.4)	6.6 (11.8)	6.5 (11.2)	9.9 (7.1)	.001
Age group, y					
0-1	160 (23.4)	101 (23.6)	55 (28.1)	4 (6.2)	.002
2-5	137 (20.0)	93 (21.7)	35 (18.2)	9 (13.8)	
6-10	127 (18.5)	74 (17.3)	33 (17.2)	20 (30.8)	
>10	261 (38.1)	160 (37.4)	69 (35.9)	32 (49.2)	
Male	386 (56.4)	239 (55.8)	111 (57.8)	36 (55.4)	3
Female	299 (43.6)	189 (44.2)	81 (42.2)	29 (44.6)	
Coexisting conditions	121 (17.7)	83 (19.4)	34 (17.7)	4 (6.2)	.03
Neurological and psychiatric disease	32 (4.7)	20 (4.7)	11 (5.7)	1 (1.5)	.38
Oncohematological disease	19 (2.8)	16 (3.7)	3 (1.6)	0	.26
Endocrinological disease	15 (2.2)	7 (1.6)	7 (3.6)	1 (1.5)	.27
Genetic/metabolic disease	13 (1.9)	10 (2.3)	3 (1.6)	0	.80
Cardiological disease	13 (1.9)	7 (1.6)	5 (2.6)	1 (1.5)	.70
Immunorheumatological disease	11 (1.6)	7 (1.6)	3 (1.6)	1 (1.5)	.10
Chronic kidney disease	10 (1.5)	9 (2.1)	1 (0.5)	0	.35
Gastrointestinal disease	8 (1.2)	7 (1.6)	1 (0.5)	0	.52
Immunosuppression	14 (2.0)	9 (2.1)	4 (2.1)	1 (1.5)	>.99
Diagnosis and clinical course					
Acute symptomatic SARS-CoV-2 infection	628 (91.7)	415 (97.0)	180 (93.8)	33 (50.7)	<.001
Multisystem inflammatory syndrome	57 (8.3)	13 (3.0)	12 (6.3)	32 (49.2)	<.001
Hospital					
Admission	402 (58.7)	215 (50.2)	122 (63.5)	65 (100)	<.001
Length of stay, median (IQR), d	6 (4-11)	5 (3-10)	6 (4-11)	10 (6-18)	<.001
Intensive care unit admission	41 (6.0)	13 (3.0)	9 (4.7)	19 (29.2)	<.001
Clinical signs and symptoms					
Fever	575 (83.9)	371 (86.7)	158 (82.3)	46 (70.8)	.004
Highest temperature registered, mean (SD), °C	38.55 (0.74)	38.38 (0.66)	38.7 (0.74)	39.1 (0.79)	<.001
Respiratory symptoms					
Cough	258 (37.7)	182 (42.5)	72 (37.5)	4 (6.2)	<.001
Rhinorrhea	147 (21.5)	107 (25.0)	37 (19.3)	3 (4.6)	.001
Pharyngitis	88 (12.8)	56 (13.1)	25 (13.0)	7 (10.8)	.87
Dyspnea	62 (9.1)	39 (9.1)	18 (9.4)	5 (7.7)	.92
ARDS	8 (1.2)	3 (0.7)	3 (1.6)	2 (3.1)	.21
Chest radiograph performed	303 (44.2)	174 (40.7)	87 (45.3)	42 (64.6)	
Pathologic	160 (52.8)	103 (59.2)	46 (52.9)	21 (50.0)	.41
Lobar	33 (10.9)	18 (10.3)	9 (10.3)	6 (14.3)	
Interstitial	82 (27.1)	43 (24.7)	26 (29.9)	13 (31.0)	
Both	45 (14.9)	32 (18.4)	11 (12.6)	2 (4.8)	
Gastrointestinal symptoms					
Diarrhea	131 (19.1)	NA	107 (55.7)	24 (36.9)	.009
Vomiting	97 (14.2)	NA	58 (30.2)	39 (60.0)	<.001
Abdominal pain	96 (14.0)	NA	39 (20.3)	57 (87.7)	<.001
Other signs and symptoms					
Anorexia/nausea	85 (12.4)	5 (1.2)	55 (28.6)	25 (38.5)	<.001
Dysgeusia/anosmia	33 (4.8)	19 (4.4)	12 (6.3)	2 (3.1)	.49
Conjunctivitis	62 (9.1)	33 (7.7)	20 (10.4)	9 (13.8)	.20
Cardiac involvement	44 (6.4)	6 (1.4)	8 (4.2)	30 (46.2)	<.001
Laboratory findings					
Leukocytosis^b^	96/462 (20.8)	34/253 (13.4)	33/144 (22.9)	29/65 (44.6)	<.001
Lymphopenia^b^	106/457 (23.2)	38/253 (15.0)	25/144 (17.4)	43/60 (71.7)	<.001
Increased CRP	216/459 (47.1)	96/250 (38.4)	62/144 (43.1)	58/65 (89.2)	<.001
Increased LDH	82/372 (22.0)	38/201 (18.9)	27/117 (23.1)	17/54 (31.5)	.13
Increased ferritin	70/236 (29.7)	20/109 (18.3)	14/76 (18.4)	36/51 (70.6)	<.001
Increased ALT	60/432 (13.9)	24/236 (10.2)	23/136 (16.9)	13/60 (21.7)	.03
Diagnosis of SARS-CoV-2 infection					
Positive^c^					
Nasopharyngeal swab	635 (92.7)	408 (95.3)	181 (94.3)	46 (70.8)	<.001
IgM/IgG antibodies	56 (8.2)	20 (4.7)	15 (7.8)	21 (32.3)	.42
Other laboratory-confirmed infections	77/267 (28.8)	32/136 (23.5)	31/79 (39.2)	14/52 (26.9)	.047
Treatment					
Antibiotic therapy	224 (32.7)	102 (23.8)	65 (33.9)	57 (87.7)	<.001
Antiviral therapy	21 (3.1)	14 (3.3)	7 (3.6)	0	.60
Hydroxychloroquine	53 (7.7)	34 (7.9)	17 (8.9)	2 (3.1)	.31
Intravenous immunoglobulins	22 (3.2)	5 (1.2)	9 (4.7)	8 (12.3)	<.001
Systemic corticosteroids	22 (3.2)	8 (1.9)	9 (4.7)	5 (7.7)	.02
Anti-IL-1, IL-6 monoclonal antibodies	6 (0.9)	0	1 (0.5)	5 (7.7)	<.001

Abbreviations: ALT, alanine aminotransferase; ARDS, acute respiratory distress syndrome; CRP, C-reactive protein; GI, gastrointestinal; Ig, immunoglobulin; IL, interleukin; LDH, lactic dehydrogenase; NA, not applicable.

^a^ *P* < .05 was considered statistically significant.

^b^ Leukocyte and lymphocyte values were compared with reference range values according to age ranges.

^c^ Few patients underwent serologic testing, mainly during the first pandemic wave. The sum of microbiological tests in each category is superior to the total of patients, as some of them contemporarily presented positive nasopharyngeal swab and IgM/IgG antibodies.

Two-hundred fifty-seven (37.5%) children showed GI symptoms during the disease course. The presence of GI symptoms was reported most often in hospitalized children compared with those who were outpatients (293 [72.9%] vs 78 [27.6%]; *P* < .001) and was associated with a higher chance of hospital admission (OR, 2.64; 95% CI, 1.89-3.69) and intensive care admission (OR, 3.90; 95% CI, 1.98-7.68).

One-hundred ninety-two (74.7%) children presented with mild to moderate GI involvement characterized by diarrhea (107 [55.71%]) in most cases, vomiting (58 [30.2%]), or abdominal pain (39 [20.3%]) (Table 1). Among the 57 children with a diagnosis of MIS-C, 44 (77.2%) showed evidence of GI involvement.

An intestinal pathogen was isolated in 24 children, specifically, rotavirus (n = 12), *Campylobacter* species (n = 5), adenovirus (n = 4), *Salmonella* species (n = 1), enteropathogenic *Escherichia coli* (n = 1), and *Enterobius vermicularis* (n = 1). Notably, *E vermicularis* was identified as a potential etiological trigger of appendicitis in the child.

### Factors Associated With Severe GI Manifestations

Sixty-five children (9.5%) showed clinically severe GI involvement (Table 2), including disseminated adenomesenteritis (39.6%), appendicitis (33.5%), abdominal fluid collection (21.3%), pancreatitis (6.9%), or intussusception (4.6%). All of those children were hospitalized and had a longer median hospital stay (10 [IQR, 6-18] days) and an increased risk of intensive care unit admission (19 [29.2%]) compared with those with mild to moderate (9 of 192 [4.7%]) or no (13 of 428 [3.0%]) GI symptoms (*P* < .001) (Table 1).

**Table 2.** Clinical and Radiological Findings in 65 Children With COVID-19 Presenting With Severe and Atypical GI Manifestations

GI manifestations	Cases, No. (%)
No.	65
Mesenteric fat inflammation	47 (72.3)
Intestinal wall thickening	44 (67.7)
Peritoneal effusion	42 (64.6)
Abdominal lymphadenopathy	39 (60.0)
Appendicitis	
Clinically suspected	33 (50.8)
Complicated by perforation	5 (7.7)
Complicated by peritonitis	3 (4.6)
Undergoing surgery	23 (35.4)
Abdominal abscesses/fluid collections	21 (32.3)
Pancreatitis	6 (9.2)
Ileal or ileo-colic intussusception	4 (6.2)

Abbreviation: GI, gastrointestinal.

Abdominal pain (57 [87.7%]) and vomiting (39 [60%]) were the more frequently reported symptoms in this subgroup; conversely, fever, cough, or rhinorrhea were less likely reported in comparison with children with SARS-CoV-2 infection children without GI involvement (Table 1).

**Figure 1.**
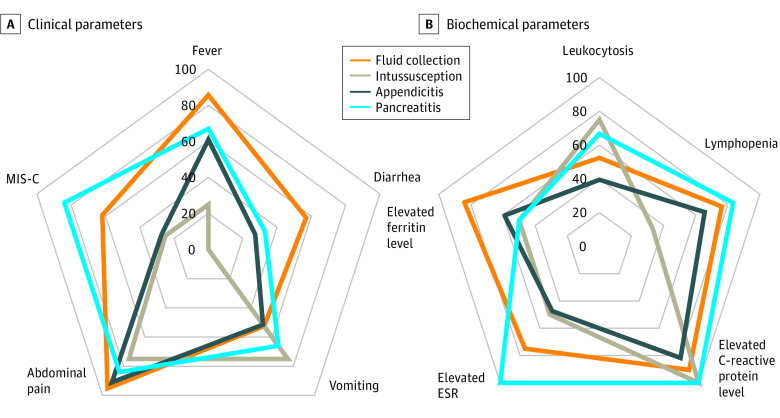
Clinical and Biochemical Presentation of Children With Different Gastrointestinal Manifestations Abbreviations: ESR, erythrocyte sedimentation rate; MIS-C, multisystem inflammatory syndrome in children.

The clinical and biochemical presentation varied, according to each GI scenario (Figure 1). Children with intussusception presented with abdominal pain and vomiting, but rarely had fever, diarrhea, or received a diagnosis of MIS-C; conversely, pancreatitis and abdominal fluid collection were more frequently observed in patients with MIS-C and presented with pain, fever, and vomiting. The markers of inflammation were increased in most conditions.

Age was a relevant risk factor for severe GI presentation. Children with severe GI symptoms were older (9.9 [7.1] years) than both those with mild to moderate (6.5 [11.2] years) or no (6.6 [11.8] years) GI symptoms (*P* = .001) (Table 1). In addition, children aged 5 to 10 years (OR, 8.33; 95% CI, 2.62-26.5) or older than 10 years (OR, 6.37; 95% CI, 2.12-19.1) had a higher chance of severe outcomes in comparison with infants (eTable 1 in Supplement 1).

The presence of any underlying chronic condition was not associated with an increased chance of severe GI manifestation. None of children with chronic GI diseases (n = 8) had severe GI manifestations during COVID-19 or developed MIS-C (Table 1).

Compared with those with mild to moderate GI features, children presenting with abdominal pain (OR, 27.9; 95% CI, 12.32-63.4), vomiting (OR, 3.47; 95% CI, 1.93-6.21), leukocytosis (OR, 2.83; 95% CI, 1.51-5.28), lymphopenia (OR, 11.2; 95% CI, 5.63-22.4), elevated C-reactive protein levels (OR, 11.6; 95% CI, 4.97-27.1), or increased ferritin levels (OR, 10.62; 95% CI, 4.61-24.52) had an increased chance of severe GI involvement in univariate analysis (eTable 1 in Supplement 1). The incidence of these findings is reported in Table 2. This clinical and biochemical presentation was independent from the presence of coinfections and similar for different severe GI manifestations. However, among children with severe GI presentation, higher levels of leukocytes (mean [SD], 14 600 [5250] vs 11 500 [4900]/μL; *P* = .03 [to convert to ×10^9^ per liter, multiply by 0.001]), C-reactive protein (22.0 [10.1] vs 9.59 [11.6] mg/dL; *P* < .001 [to convert to milligrams per liter, multiply by 10]), and ferritin (804.8 [440.1] vs 328.1 [238.1] ng/mL; *P* < .001 [to convert to micrograms per liter, multiply by 1]) were observed in children with MIS-C compared with children with SARS-CoV-2 infection.

Most of the 57 children with MIS-C (32 [56.1%]) had severe GI involvement, and conversely, MIS-C accounted for about half of children with severe GI manifestations and for all 6 cases of pancreatitis. In univariate analysis, MIS-C was associated with a higher risk of appendicitis (OR, 4.71; 95% CI, 2.07-10.7), abdominal fluid collection (OR, 22.9; 95% CI, 9.0-58.1), adenomesenteritis (OR, 24.3; 95% CI, 11.8-49.7), and pancreatitis (OR, 60.3; 95% CI, 6.9-525.7) (eTable 1 in Supplement 1). Among children who received a diagnosis of appendicitis, all of those with concomitant MIS-C had an increased level of C-reactive protein compared with 74% of children without MIS-C (mean [SD], 16.8 [12.0] vs 4.8 [4.10] mg/dL; *P* = 0.008), although the levels of leukocytes, lymphocytes, and ferritin were similar. As expected, children with severe GI manifestations more frequently received antibiotic and anti-inflammatory treatment compared with those with no or mild to moderate GI symptoms (Table 1).

**Figure 2.**
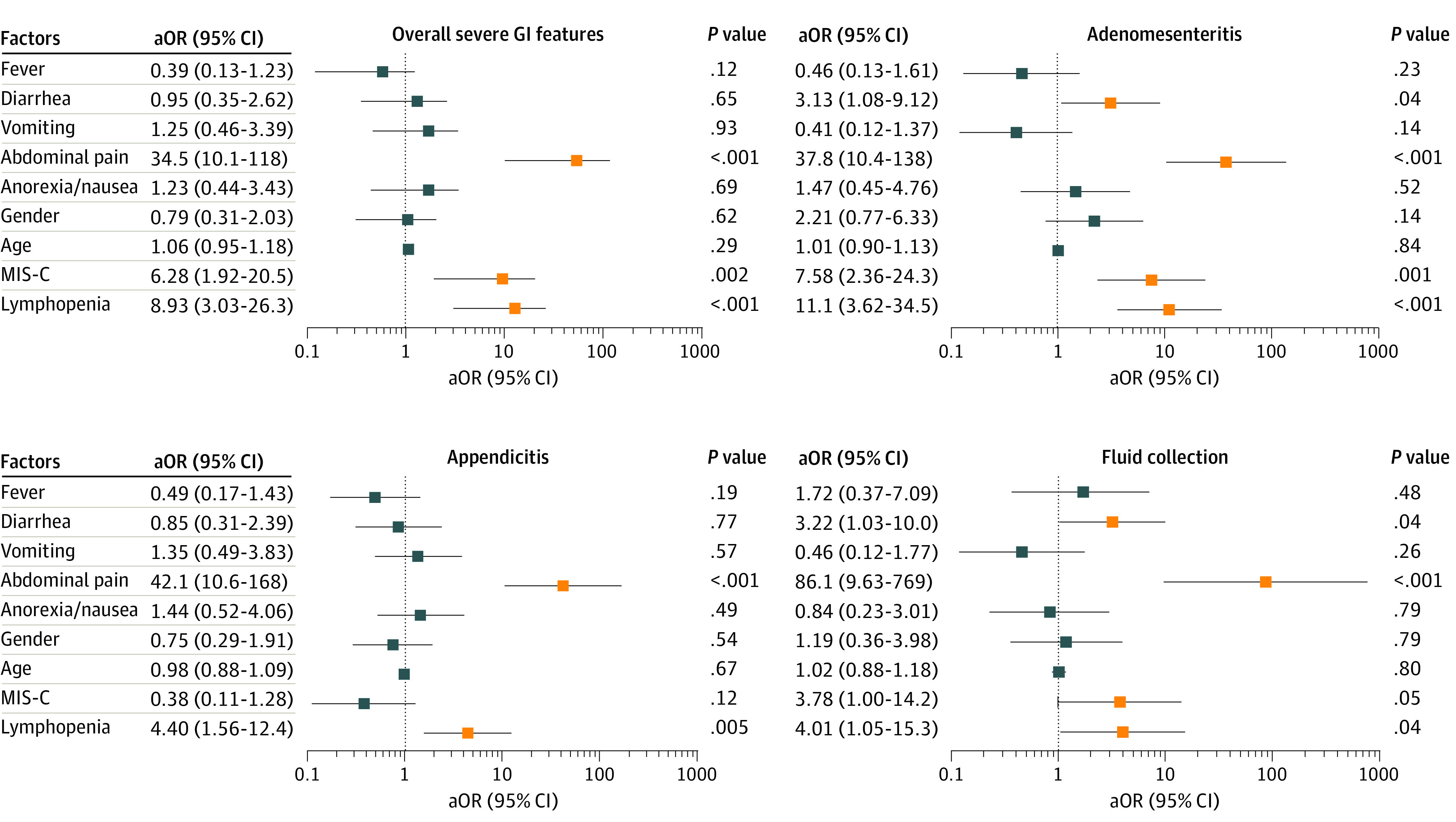
Factors Associated With Severe Gastrointestinal (GI) Outcomes in Multivariable Analysis Adjusted odds ratios (aORs) and 95% CIs of factors associated with severe GI manifestations, adenomesenteritis, appendicitis, and fluid collection. Multivariable analysis included age, sex, GI symptoms, multisystem inflammatory syndrome in children (MIS-C), and variables found to have *P* ≤ .10 in the univariate analysis (eTable 1 in Supplement 1). Parameters necessary for the definition of MIS-C (ie, elevated leukocyte, C-reactive protein, and ferritin levels) were excluded from multivariable analysis to avoid biases. Red lines indicate significant findings; whiskers, 95% CIs.

In multivariable analysis, severe GI manifestations were associated with abdominal pain (aOR, 34.5; 95% CI, 10.1-118), lymphopenia (aOR, 8.93; 95% CI, 3.03-26.3), or MIS-C (aOR, 6.28; 95% CI, 1.92-20.5). Diarrhea was associated with a higher chance of adenomesenteritis (aOR, 3.13; 95% CI, 1.08-9.12) or abdominal fluid collection (aOR, 3.22; 95% CI, 1.03-10.0) (Figure 2; eTable 2 in Supplement 1).

### Radiological Findings

All but one child underwent abdominal ultrasonography (52 [80.0%]) or tomography (12 [18.5%]) evaluation. One child presented with acute abdomen and underwent urgent surgery without imaging. The majority of patients showed a radiological feature characterized by diffuse peritoneal effusion, mesenteric fat inflammation, multiple mesenteric lymphadenopathies, or intestinal wall thickening (Table 2). In children receiving a clinical diagnosis of appendicitis, imaging generally confirmed the presence of peritoneal effusion and appendicular thickening, although the median appendicular diameter (when available) was 5 mm.

**Figure 3.**
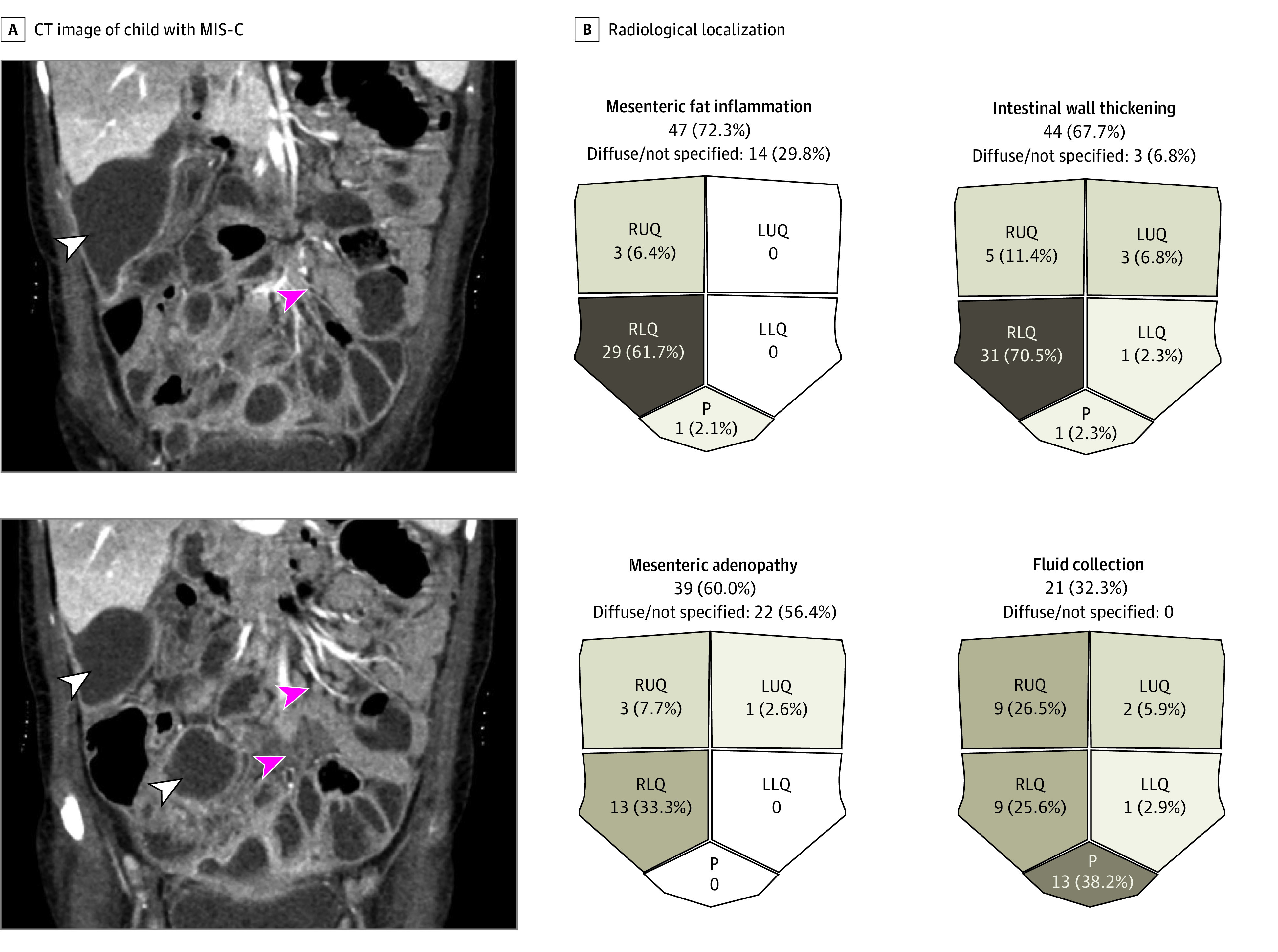
Imaging Findings in Children With Severe Gastrointestinal (GI) Involvement A, Abdominal computed tomography (CT) images of a child (age, 4 years) affected by multisystem inflammatory syndrome in children (MIS-C) with subhepatic and diffuse mesenteric fluid collection (red arrowheads), mesenterial adenopathy, and fat inflammation (white arrowheads). B, Radiological localization of the most frequent GI manifestations. Fluid was collected from more than 1 site in some patients (ie, 34 sites in 21 patients). LLQ indicates left lower quadrant; LUQ, left upper quadrant; P, pelvis; RLQ, right lower quadrant; and RUQ, right upper quadrant.

Twenty-one patients (32.3%) showed single or multiple uneven fluid collection frequently localized in the lower right quadrant or subhepatic and retrovesical area, and was usually referred to as abdominal abscesses (Figure 3A). Figure 3B depicts the frequency and localization of most reported radiological findings.

### Findings in Children Undergoing Surgery

Twenty-seven children (41.5%) underwent surgery. Twenty-three children (35.4%) received a clinical and radiological diagnosis of acute appendicitis, with 8 complicated by peritonitis or intestinal perforation (Table 2). Reports on pathologic findings were available for 15 children, and among them only 9 (60.0%) had a histologically confirmed diagnosis of acute appendicitis.

Nine patients with MIS-C received a clinical diagnosis of appendicitis, but none of these was histologically confirmed. Microscopic analysis of specimens from the 4 children with MIS-C who underwent surgery showed a normal appendix (n = 2) or an isolated perivisceritis (n = 1) or serositis (n = 1) with vascular congestion (n = 2) not supporting the initial clinical diagnosis (eFigure 2 in Supplement 1).

Four of 27 children underwent surgery for reasons other than appendicitis: 2 infants with ileocolic intussusception, 1 child with adenomesenteritis and a solid mass needing excisional biopsy, and a child aged 4 years with MIS-C and multiple abdominal collections (Figure 3A) who developed ileum secondary to ab-extrinseco obstruction.

Most abdominal fluid collections had a resolution over time without surgery (data on timing not available). Drainage was performed in only 5 of 21 children (23.8%) in the context of abdominal laparoscopy or open surgery for suspected appendicitis or intestinal obstruction, based on local medical indications. In all these children, no bacterial growth was observed.

## Discussion

In this large cohort of Italian children with COVID-19, we observed GI symptoms in more than one-third of the patients. Most children with GI involvement had benign and self-limiting symptoms comparable to those observed in other viral intestinal infections. However, a subset of children developed severe GI manifestations characterized by diffuse adenomesenteritis, abdominal fluid collection, appendicitis, ileal intussusception, or pancreatitis. Similar manifestations have been sporadically reported during the pandemic^4^; however, the frequency, clinical course, and outcome of these conditions are still unknown. Moreover, and in line with previous evidence,^8,9^ we observed that a clinical presentation with GI symptoms was associated with a higher chance of hospitalization and intensive care support.

In the current scenario, characterized by an increase in COVID-19 cases^13^ and, at the time of the study, limited access to vaccination in the pediatric age group, identifying the factors related to severe GI involvement represents a scientific unmet need and may provide supporting information to practitioners working in emergency department and primary care settings. Overall, our data show a prevalence of GI symptoms among the highest reported in literature.^4,17,18^ In our cohort of inpatients and outpatients, 37.5% of children with COVID-19 presented with at least 1 GI symptom.

Owing to the abundant expression of binding receptors (angiotensin-converting enzyme 2 and transmembrane serine protease 2) on the surface of enterocytes,^1,2^ SARS-CoV-2, similarly to other coronaviruses,^19^ has a direct action on enterocytes, suggesting the need to include it among the differential diagnosis of acute diarrhea, vomiting, and abdominal pain.^4^

Herein, we report that 9.5% of children with symptomatic SARS-CoV-2 infection may develop atypical and clinically severe GI manifestations that require hospitalization. Those features are related to the child’s age (>5 years) but not to the presence of underlying conditions or the severity of respiratory symptoms.

A clinical presentation characterized by abdominal pain, lymphopenia, and increased C-reactive protein and ferritin levels was associated with a 9- to 30-fold increased probability of severe GI outcomes. Similarly, children fulfilling the criteria of MIS-C had a higher chance of receiving a diagnosis of adenomesenteritis or abdominal fluid collections. These findings identify the GI tract as a potential target of the immune-mediated inflammatory response triggered by SARS-CoV-2, of which MIS-C represents the capital manifestation usually associated with the highest degree of inflammation. About half of the patients with severe GI involvement underwent acute surgical intervention, most following a diagnosis of intussusception, diffuse fluid collection, or a clinical feature previously described as appendicitis or pseudoappendicitis.^11,12,20,21^

In multivariable analysis, appendicitis was associated with abdominal pain and lymphopenia but not with MIS-C; notably, 40.0% of the diagnoses were not histologically confirmed. This high percentage of histologically proven negative cases in children with COVID-19 contrasts with previous evidence reporting less than 20% of unconfirmed diagnoses in children undergoing appendectomy before the pandemic era.^22,23^

Consistent with previous evidence,^24^ in some of the children in this report, SARS-CoV-2 might have triggered appendicitis with a typical clinical and histologic feature and an expected complication rate of 10% to 20%. However, children with MIS-C showing appendicitislike symptoms had a low rate of complications and histologic findings of serositis and perivisceritis in the absence of intraluminal obstruction.

Lishman et al^25^ reported few cases of appendicitis in children with MIS-C and suggested inflammation or vasculitis as a pathogenic mechanism owing to the lack of lumen obstruction and fecoliths. In patients with MIS-C, the cytokine-mediated inflammation may affect lymph nodes, fat throughout the mesentery, and peritoneum, also involving the intestinal wall and appendix with a probable serosa-to-lumen path. This pattern may be induced by artery vasculitis, similar to what is reported in Kawasaki disease.^4,26^

During the first pandemic wave, Tullie et al^10^ described a severe GI feature “which might be mistaken for appendicitis” in a small series of children with MIS-C who did not require surgery. This hypothesis needs to be further explored by comparative histologic studies. However, the absence of a statistically shown association between MIS-C and appendicitis in our cohort and the resolution of most cases with medical treatment^10^ may support a more conservative approach in children presenting with acute abdomen and MIS-C.

Fluid collections were identified in a subgroup of patients and likely reflect the delimitation of inflammatory fluids throughout the omentum. In multivariable analysis, the presence of diarrhea, abdominal pain, or MIS-C was associated with the finding of abdominal fluid collection, usually referred to as abscesses. Although increased intestinal permeability and bacterial translocation could be hypothesized, a thorough search of bacteria in patients who underwent surgery yielded consistently negative results. This finding, together with a frequent spontaneous resolution, supports the hypothesis of an inflammatory but noninfectious pathogenesis.

Intussusception was found in 4 children presenting with gelatinous or loose stools, vomiting, and abdominal pain. This complication was not associated with the child’s age (although it was more common in those aged <1 year), lymphopenia, or MIS-C. Conclusions cannot be made in such a small population. The paucity of cases reported so far^27^ may suggest that SARS-CoV-2, even rarely, can act as a trigger for intestinal intussusception, as demonstrated for other enteric viruses.^28^

### Strengths and Limitations

This study has limitations, with the retrospective design being the major limitation. Frequency of diarrhea or vomiting, the characteristics of stools, or the degree of pain were not specifically recorded, resulting in scattered and incomplete data that do not allow clear distinction between mild and moderate symptoms or accurately describe the course of symptoms. To limit reporting bias, we provided a stringent definition of severe GI manifestation and contacted single study participants to retrieve detailed information about the primary outcomes. We believe that the discussion with peers and the review of clinical reports by 2 independent investigators supports a reliable and accurate definition of the primary outcome. The interpretation of histologic findings was based on local medical reports because a centralized reassessment of the specimens was not feasible. However, when needed, a blinded pathologist reconsidered single histologic reports.

The case report form did not include information on timing for most clinical findings and laboratory tests. Thus, we cannot rule out the possibility that some parameters might be markers, rather than estimators of probability, of severe outcomes.

Potential differences in patients’ management among health care institutions could have affected findings. However, as members of the SITIP network, most investigators shared information during their weekly meetings and developed joint national recommendations.^29^ This practice supported homogeneity in management protocols and strengthened the study results. In addition, the enrollment of children in outpatient and inpatient settings from areas with a different pandemic burden may provide reliable and generalizable results that depict the overall GI presentation and threats of children with acute COVID-19 and MIS-C.

## Conclusions

In this multicenter cohort study of Italian children with SARS-CoV-2 infection and MIS-C, we observed that GI symptoms were more frequent than in other reports at that time and suggest that approximately 1 of 10 children with COVID-19 may develop severe GI manifestations requiring high rates of hospitalization and intensive care unit admission.

Awareness about the factors associated with severe GI manifestations may help practitioners working either in emergency department or primary care settings to identify children with these diagnoses and manage children at risk for severe outcomes. A high grade of suspicion should be maintained in school-aged children and adolescents as well as all children presenting with abdominal pain, leukopenia, and elevated inflammatory markers or MIS-C who may require rapid abdominal imaging and surgical consultation.

## Notes

Supplement 1. **eFigure 1.** Flow Diagram of Study Population Enrollment **eTable 1.** Univariable Analysis of Factors Associated With Severe GI Outcome and Single GI Manifestations **eTable 2.** Multivariable Analysis of Factors Associated With Severe GI Outcomes **eFigure 2.** Histological Findings of a Child With MIS-C Undergoing Surgery for Clinical Features of Acute Appendicitis

Supplement 2. **Nonauthor Collaborators.** The Italian SITIP-SIP Paediatric SARS-CoV-2 Infection Study Group
