# Supplementary material for: Factors Associated With Severe Gastrointestinal Diagnoses in Children With SARS-CoV-2 Infection or Multisystem Inflammatory Syndrome
Source: JAMA Netw Open. 2021 Dec 20;4(12):e2139974. doi: 10.1001/jamanetworkopen.2021.39974 (PMC8689385; doi:10.1001/jamanetworkopen.2021.39974)
Supplement: Supplement 1. — eFigure 1. Flow Diagram of Study Population Enrollment eTable 1. Univariable Analysis of Factors Associated With Severe GI Outcome and Single GI Manifestations eTable 2. Multivariable Analysis of Factors Associated With Severe GI Outcomes eFigure 2. Histological Findings of a Child With MIS-C Undergoing Surgery for Clinical Features of Acute Appendicitis [file jamanetwopen-e2139974-s001.pdf]

Supplementary Online Content

Lo Vecchio A, Garazzino S, Smarrazzo A, et al; Italian SITIP-SIP Paediatric SARS-CoV-2 Infection Study Group. Factors associated with severe gastrointestinal diagnoses in children with SARS-CoV-2 infection or multisystem inflammatory syndrome. *JAMA Netw Open*. 2021;4(12):e2139974. doi:10.1001/jamanetworkopen.2021.39974

eFigure 1. Flow Diagram of Study Population Enrollment

eTable 1. Univariable Analysis of Factors Associated With Severe GI Outcome and Single GI Manifestations

eTable 2. Multivariable Analysis of Factors Associated With Severe GI Outcomes

eFigure 2. Histological Findings of a Child With MIS-C Undergoing Surgery for Clinical Features of Acute Appendicitis

This supplementary material has been provided by the authors to give readers additional information about their work.

© 2021 Lo Vecchio A et al. *JAMA Network Open.*

eFigure. Flow diagram of study population enrollment.

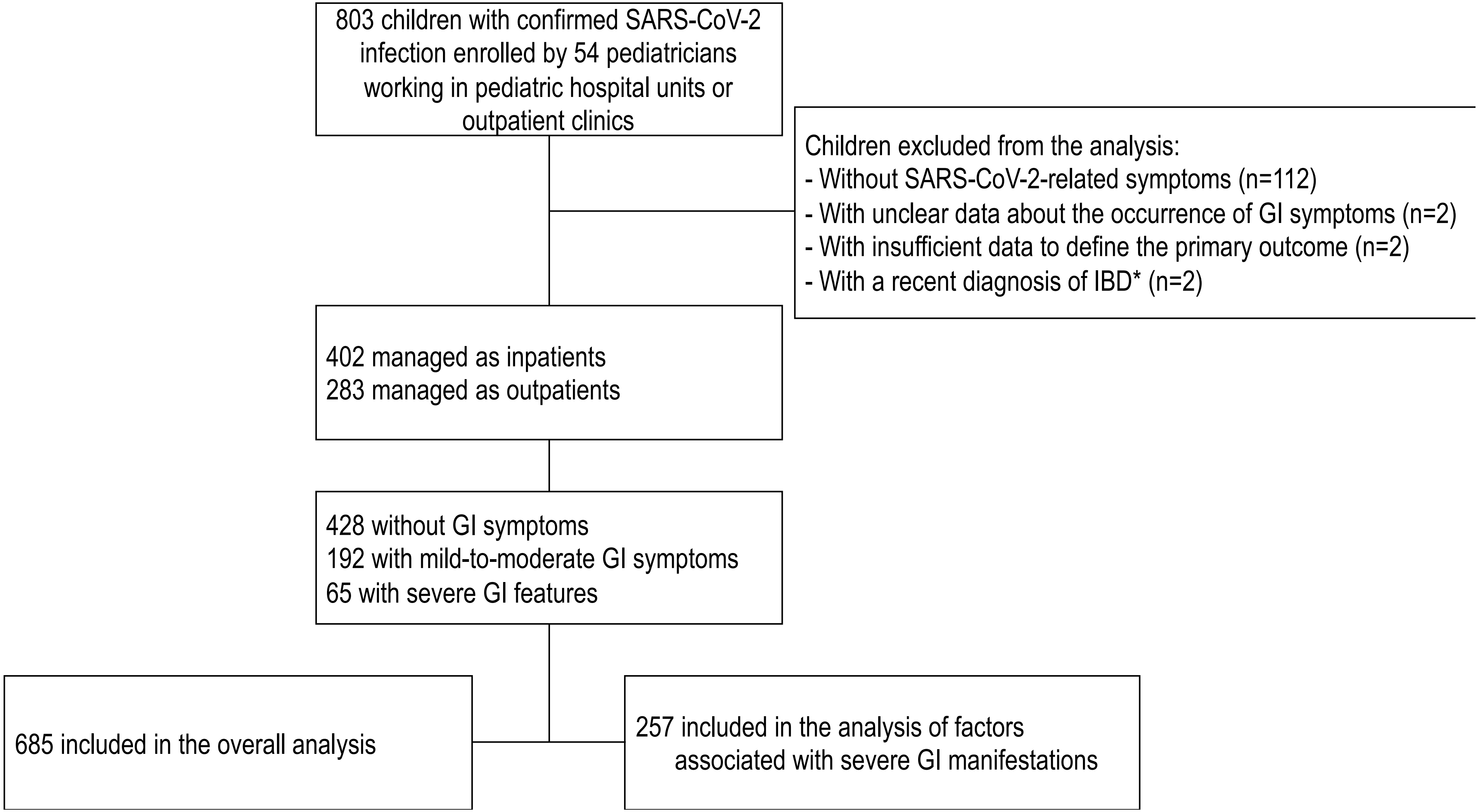

© 2021 Lo Vecchio A et al. *JAMA Network Open.*

Analysis of factors associated to severe gastrointestinal (GI) involvement does not include children without GI symptoms.
Two children received a diagnosis Inflammatory Bowel Diseases (IBD) few days/weeks before demonstrating SARS-CoV-2 infection. For these patients, it was not possible to understand if the presence and characteristics of GI symptoms were attributable to SARS-CoV-2 infection or to the disease progression or first-line treatment failure. Those patients were excluded from analysis.

© 2021 Lo Vecchio A et al. *JAMA Network Open.*

eTable 1. Univariable analysis of factors associated with severe GI outcome and single GI manifestations

Risk factor	Severe GI features OR (95%CI)	p	Adeno-mesenteritis OR (95%CI)	p	Appendicitis OR (95%CI)	p	Fluid collections OR (95%CI)	p	Intussusception OR (95%CI)	p	Pancreatitis OR (95%CI)	p
Male gender	0·90 (0·51–1·59)	0·732	1·87 (0·935–3·74)	0·073	0·92 (0·46–1·87)	0·830	1·57 (0·63–3·94)	0·333	0·77 (0·10–5·52)	0·797	1·55 (0·28–8·57)	0·609
Age < 1 year	–		–		–		–		–		–	
1 – 5 years	3·53 (1·01–12·36)	0·048	6·02 (0·69–52·2)	0·103	3·56 (0·366–34·6)	0·274	1·17 (0·72–18·9)	0·912	0·58 (0·05–6·47)	0·659	1	1·000
5 – 10 years	8·33 (2·62–26·5)	0·000	16·5 (2·1–129·4)	0·007	13·6 (1·72–107·6)	0·013	12·1 (1·5–97·0)	0·019	0·63 (0·05–6·99)	0·704	NA	NA
>10 years	6·37 (2·12–19·1)	0·001	14·6 (1·95–109·7)	0·009	12·4 (1·65–94·2)	0·014	6·33 (0·80–49·9)	0·080	NA	0·995	NA	NA
Underlying conditions	0·30 (0·10–0·89)	0·031	0·11 (0·01–0·82)	0·010	0·45 (0·13–1·50)	0·186	0·22 (0·30–1·70)	0·115	NA	NA	2·35 (0·43–12·9)	0·321
GI symptoms												
Diarrhea	2·15 (1·20–3·83)	0·009	4·30 (2·24–8–27)	0·000	1·62 (0·74–3·59)	0·222	6·10 (2·52–14·8)	0·000	NA	NA	2·13 (0·38–11·7)	0·374
Vomiting	3·47 (1·93–6·21)	0·000	8·27 (4·25–16·0)	0·000	7·59 (3·70–15·6)	0·000	7·39 (3·05–17·9)	0·000	18·7 (1·92–181·9)	0·000	12·6 (2·27–69–7)	0·000
Abdominal pain	27·9 (12·32–63·4)	0·000	67·0 (25·3–177–4)	0·000	88·7 (26·3–298·0)	0·000	154·7 (20·4–1169·4)	0·000	18·9 (1·95–184·3)	0·000	32·3 (3·7–279·7)	0·000
Anorexia/nausea	1·55 (0·86–2·80)	0·141	4·35 (2·17–8·72)	0·000	4·53 (2·14–9·59)	0·000	3·75 (1·47–9·59)	0·003	2·37 (0·244–23·0)	0·444	3·59 (0·65–19·9)	0·118
Fever	0·52 (0·27–0·99)	0·049	0·55 (0·26–1·16)	0·112	0·27 (0·12–0·56)	0·000	1·15 (0·33–3·98)	0·822	0·06 (0·006–0·60)	0·001	0·37 (0·06–2·09)	0·247
MIS-C	14·5 (6·80–31·1)	0·000	24·3 (11·8–49·7)	0·000	4·71 (2·07–10·7)	0·000	22·9 (9·0–58·1)	0·000	3·72 (0·38–36·4)	0·226	60·3 (6·9–525·7)	0·000
Leukocytosis	2·83 (1·51–5·28)	0·001	2·04 (1·01–4·13)	0·043	2·81 (1·34–5·88)	0·004	4·77 (1·96–11·6)	0·000	12·2 (1·25–118·5)	0·006	8·19 (1·47–45·4)	0·004
Lymphopenia	11·2 (5·63–22·4)	0·000	19·35 (8·57–43·7)	0·000	7·97 (3·70–17·1)	0·000	12·8 (4·58–35·9)	0·000	1·73 (0·15–19·3)	0·651	18·0 (2·08–156–4)	0·000
Elevated CRP	11·6 (4·97–27·1)	0·000	17·4 (5·29–57–4)	0·000	5·95 (2·40–14·7)	0·000	12·2 (2·82–53·2)	0·000	NA	NA	NA	NA
Elevated Ferritin	10·62 (4·61–24·52)	0·000	9·39 (4·32–20·5)	0·000	3·97 (1·61–9·80)	0·002	16·1 (4·51–57·4)	0·000	2·39 (0·15–38·7)	0·527	2·43 (0·47–12·3)	0·269
Co-infection	0·57 (0·26–1·22)	0·146	0·94 (0·43–2·06)	0·880	0·75 (0·26–2·14)	0·596	1·03 (0·35–3·09)	0·957	0·82 (0·08–8·00)	0·864	0·48 (0·05–4·24)	0·506

© 2021 Lo Vecchio A et al. *JAMA Network Open.*

eTable 2. Multivariable analysis of factors associated with severe GI outcomes.

Risk factor	**Severe GI features** aOR (95%CI)	**p**	**Adeno–mesenteritis** aOR (95%CI)	**p**	**Appendicitis** aOR (95%CI)	**p**	**Fluid collections** aOR (95%CI)	**p**
**Symptoms**								
Fever	0·39 (0·127–1·23)	0·109	0·46 (0·13–1·61)	0·226	0·49 (0·17–1·43)	0·193	1·72 (0·37–7·09)	0·484
Diarrhea	0·95 (0·35–2·62)	0·649	3·13 (1·08–9·12)	0·036	0·85 (0·31–2·39)	0·765	3·22 (1·03–10·0)	0·044
Vomiting	1·25 (0·46–3·39)	0·929	0·41 (0·12–1·37)	0·144	1·35 (0·49–3·83)	0·570	0·46 (0·12–1·77)	0·263
Abdominal pain	34·5 (10·1–118·2)	0·000	37·8 (10·4–138·7)	0·000	42·1 (10·6–167·7)	0·000	86·1 (9·63–768·8)	0·000
Anorexia/nausea	1·23 (0·44–3·43)	0·694	1·47 (0·45–4·76)	0·523	1·44 (0·52–4·06)	0·488	0·84 (0·23–3·01)	0·791
**Other factors**								
Gender	0·79 (0·31–2·03)	0·623	2·21 (0·77–6·33)	0·138	0·75 (0·29–1·91)	0·543	1·19 (0·36–3·98)	0·786
Age	1·06 (0·95–1·18)	0·290	1·01 (0·90–1·13)	0·837	0·98 (0·88–1·09)	0·672	1·02 (0·88–1–18)	0·801
MIS–C	6·28 (1·92–20·5)	0·002	7·58 (2·36–24·3)	0·001	0·38 (0·11–1·28)	0·119	3·78 (1·00–14·2)	0·050
Lymphopenia	8·93 (3·03–26·3)	0·000	11·1 (3·62–34·5)	0·000	4·40 (1·56–12·4)	0·005	4·01 (1·05–15·3)	0·042

© 2021 Lo Vecchio A et al. *JAMA Network Open.*

eFigure 2. Histological findings of a child with MIS-C undergoing surgery for clinical features of acute appendicitis. Pathology Images as courtesy of Dr. Ettore Macrì – San Martino Hospital Belluno (IT).

A) H&E stain 20x: Nearly normal appendix with follicular hyperplasia, mild inflammatory infiltrate and vascular congestion.

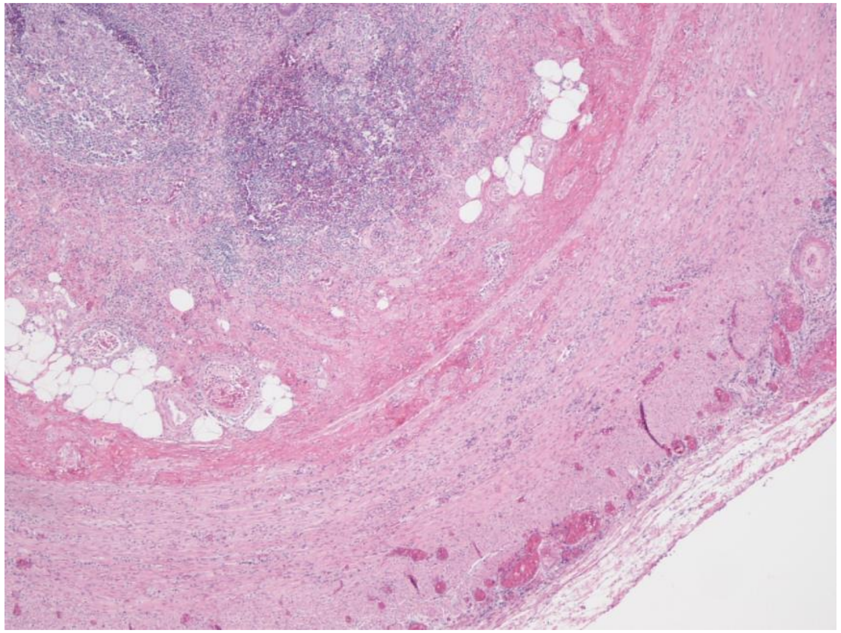

© 2021 Lo Vecchio A et al. *JAMA Network Open.*

B) H&E stain 400X: Mild and focal perivisceral neutrophilc infiltrate

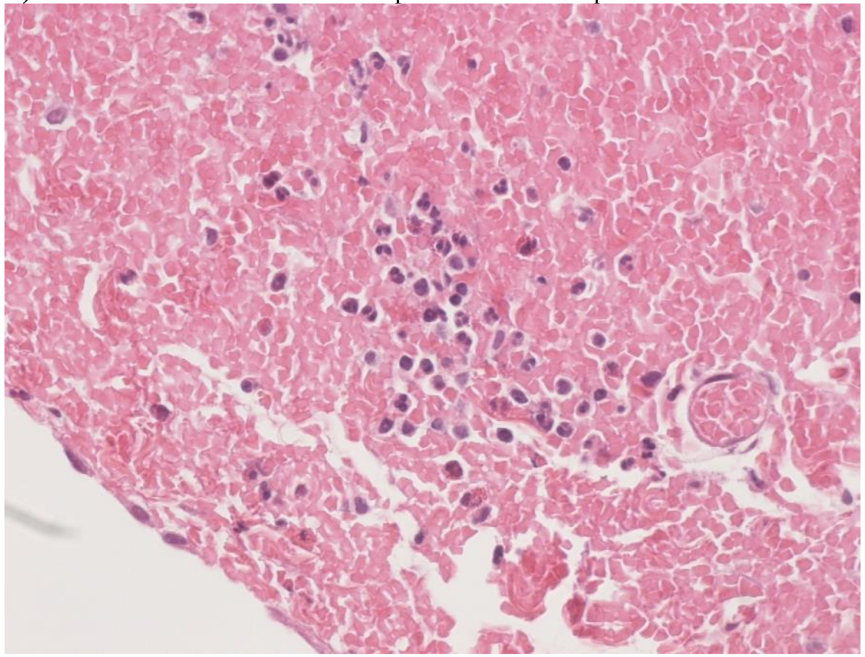

© 2021 Lo Vecchio A et al. *JAMA Network Open.*

C) H&E stain 200X: Vascular congestion

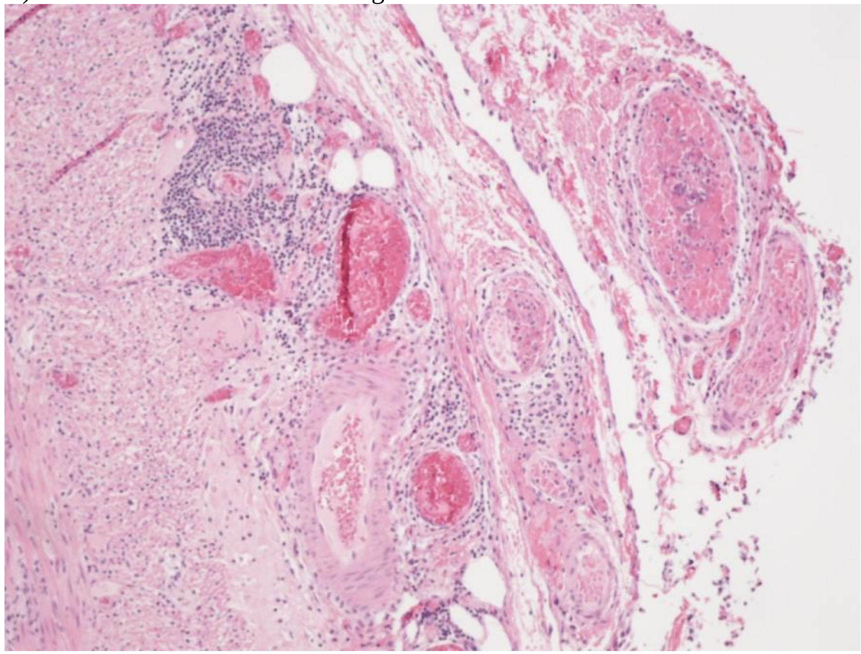

© 2021 Lo Vecchio A et al. *JAMA Network Open.*
