# Supplementary material for: Factors Associated With Severe Gastrointestinal Diagnoses in Children With SARS-CoV-2 Infection or Multisystem Inflammatory Syndrome
Source: JAMA Netw Open. 2021 Dec 20;4(12):e2139974. doi: 10.1001/jamanetworkopen.2021.39974 (PMC8689385; doi:10.1001/jamanetworkopen.2021.39974)
Supplement: Supplement 2. — Nonauthor Collaborators. The Italian SITIP-SIP Paediatric SARS-CoV-2 Infection Study Group [file jamanetwopen-e2139974-s002.pdf]

*Indicates required information. Only first name, last name, and suffix will appear in PubMed.

*Group Name(s): Italian SITIP-SIP Paediatric SARS-CoV-2 Infection Study Group							
***First Name and Middle Initial(s)**	***Last Name**	***Suffix (eg, Jr, III)**	Academic Degrees	Institution	Location (city, state/province, country)	Role or Contribution, eg, chair, principal investigator	Group (if more than 1 Group listed in the byline) and/or Subgroup (eg, Steering Committee)
Carlotta	Montagnani		MD	Infection Disease Unit, Meyer Children’s University Hospital	Florence, Italy	Collabolator	The Italian SITIP-SIP Pediatric SARS-CoV-2 Infection Study Group
Luca	Pierantoni		MD	Pediatric Emergency Unit, IRCCS AOU di Bologna	Bologna Italy	Collabolator	The Italian SITIP-SIP Pediatric SARS-CoV-2 Infection Study Group
Andrzej	Krzysztofiak		MD	Universitarian Hospital Department , Ospedale Pediatrico Bambino Gesù, IRCCS	Roma, Italy	Collabolator	The Italian SITIP-SIP Pediatric SARS-CoV-2 Infection Study Group
Daniele	Donà		MD	Division of Pediatric Infectious Disease, Department of Women’s and Children’s Health, University Hospital of Padua	Padua, Italy	Collabolator	The Italian SITIP-SIP Pediatric SARS-CoV-2 Infection Study Group
Sonia	Bianchini		MD	Department of Pediatrics, ASST Santi Paolo e Carlo Hospital	Milan, Italy	Collabolator	The Italian SITIP-SIP Pediatric SARS-CoV-2 Infection Study Group
Elio	Castagnola		MD	Infectious Diseases Unit, IRCCS Istituto Giannina Gaslini	Genova, Italy	Collabolator	The Italian SITIP-SIP Pediatric SARS-CoV-2 Infection Study Group
Marcello	Lanari		MD	Pediatric Emergency Unit, IRCCS AOU di Bologna	Bologna, Italy	Collabolator	The Italian SITIP-SIP Pediatric SARS-CoV-2 Infection Study Group
Emanuela	Peschiaroli		MD	Ospedale Pediatrico Bambino Gesù, IRCCS	Roma, Italy	Collabolator	The Italian SITIP-SIP Pediatric SARS-CoV-2 Infection Study Group

*Indicates required information. Only first name, last name, and suffix will appear in PubMed.

*First Name and Middle Initial(s)	*Last Name	*Suffix (eg, Jr, III)	Academic Degrees	Institution	Location (city, state/province, country)	Role or Contribution, eg, chair, principal investigator	Group (if more than 1 Group listed in the byline) and/or Subgroup (eg, Steering Committee)
Giovanni	Corsello		MD	Department of Maternal and Child Health, University of Palermo	Palermo, Italy	Collabolator	The Italian SITIP-SIP Pediatric SARS-CoV-2 Infection Study Group
Paola	Marchisio		MD	Fondazione IRCCS Cà Granda Ospedale Maggiore Policlinico	Milan, Italy	Collabolator	The Italian SITIP-SIP Pediatric SARS-CoV-2 Infection Study Group
Federico	Marchetti		MD	Department of Pediatrics, Santa Maria delle Croci Hospital	Ravenna, Italy	Collabolator	The Italian SITIP-SIP Pediatric SARS-CoV-2 Infection Study Group
Enrico	Felici		MD	Pediatric and Pediatric Emergency Unit, The Children Hospital, AO SS Antonio e Biagio e C. Arrigo	Alessandria, Italy	Collabolator	The Italian SITIP-SIP Pediatric SARS-CoV-2 Infection Study Group
Giulia	Pruccoli		MD	Pediatric Infectious Diseases Unit, Regina Margherita Children's Hospital, University of Turin	Turin, Italy	Collabolator	The Italian SITIP-SIP Pediatric SARS-CoV-2 Infection Study Group
Caldarelli	Valeria		MD	SC Pediatria, IRCCS AO Arcispedale Santa Maria Nuova	Reggio Emilia, Italy	Collabolator	The Italian SITIP-SIP Pediatric SARS-CoV-2 Infection Study Group
Paolo	Del Barba		MD	IRCCS San Raffaele Hospital	Milan, Italy	Collabolator	The Italian SITIP-SIP Pediatric SARS-CoV-2 Infection Study Group
Carlo	Agostoni		MD	Fondazione IRCCS Cà Granda Ospedale Maggiore Policlinico	Milan, Italy	Collabolator	The Italian SITIP-SIP Pediatric SARS-CoV-2 Infection Study Group
Chiara	Ghizzi		MD	Policlinico S.Orsola-Malpighi	Bologna, Italy	Collabolator	The Italian SITIP-SIP Pediatric SARS-CoV-2 Infection Study Group

*Indicates required information. Only first name, last name, and suffix will appear in PubMed.

***First Name and Middle Initial(s)**	***Last Name**	***Suffix (eg, Jr, III)**	Academic Degrees	Institution	Location (city, state/province, country)	Role or Contribution, eg, chair, principal investigator	Group (if more than 1 Group listed in the byline) and/or Subgroup (eg, Steering Committee)
Sara	Riscassi		MD	Pediatria Ospedale di Bolzano	Bolzano, Italy	Collabolator	The Italian SITIP-SIP Pediatric SARS-CoV-2 Infection Study Group
Ivana	Rabbone		MD	SCDU Pediatria, AOU "Maggiore della Carità"	Novara, Italy	Collabolator	The Italian SITIP-SIP Pediatric SARS-CoV-2 Infection Study Group
Carmelina	Calitri		MD	SC Pediatria, Ospedale degli Infermi	Rivoli, Italy	Collabolator	The Italian SITIP-SIP Pediatric SARS-CoV-2 Infection Study Group
Luisa	Abbagnato		MD	ASST Lariana Sant'Anna	Como, Italy	Collabolator	The Italian SITIP-SIP Pediatric SARS-CoV-2 Infection Study Group
Luciana	Parola		MD	ASST Ovest Milanese, Ospedale Fornaroli	Magenta, Italy	Collabolator	The Italian SITIP-SIP Pediatric SARS-CoV-2 Infection Study Group
Giuseppe	Banderali		MD	ASST Santi Paolo e Carlo	Milan, Italy	Collabolator	The Italian SITIP-SIP Pediatric SARS-CoV-2 Infection Study Group
Ilaria	Pacati		MD	ASST Bergamo Est	Seriate, Italy	Collabolator	The Italian SITIP-SIP Pediatric SARS-CoV-2 Infection Study Group
Simonetta	Cherubini		MD	ASST ValleOlona	Busto Arsizio, Italy	Collabolator	The Italian SITIP-SIP Pediatric SARS-CoV-2 Infection Study Group
Paola	Lippi		MD	Primary Care Pediatrician		Collabolator	The Italian SITIP-SIP Pediatric SARS-CoV-2 Infection Study Group
Andrea	Guala		MD	SC Pediatria Ospedale di Castelli	Verbania, Italy	Collabolator	The Italian SITIP-SIP Pediatric SARS-CoV-2 Infection Study Group

*Indicates required information. Only first name, last name, and suffix will appear in PubMed.

***First Name and Middle Initial(s)**	***Last Name**	***Suffix (eg, Jr, III)**	Academic Degrees	Institution	Location (city, state/province, country)	Role or Contribution, eg, chair, principal investigator	Group (if more than 1 Group listed in the byline) and/or Subgroup (eg, Steering Committee)
Rino	Agostiniani		MD	UO Pediatria, Ospedale San Jacopo	Pistoia, Italy	Collabolator	The Italian SITIP-SIP Pediatric SARS-CoV-2 Infection Study Group
Ahmad	Kantar		MD	Pediatric Unit, Policlinico San Pietro, Gruppo Ospedaliero San Donato	Ponte San Pietro-Bergamo, Italy	Collabolator	The Italian SITIP-SIP Pediatric SARS-CoV-2 Infection Study Group
Luca	Baroero		MD	SC Pediatria e Neonatologia, Ospedale Martini	Turin, Italy	Collabolator	The Italian SITIP-SIP Pediatric SARS-CoV-2 Infection Study Group
Nadia	Rossi		MD	Ospedale Clinicizzato di Chieti	Chieti, Italy	Collabolator	The Italian SITIP-SIP Pediatric SARS-CoV-2 Infection Study Group
Cristina	Dalmazzo		MD	SC Terapia Intensiva Neonatale, AO S. Croce e Carle	Cuneo, Italy	Collabolator	The Italian SITIP-SIP Pediatric SARS-CoV-2 Infection Study Group
Laura	Panigati		MD	SC Pediatria, Ospedale Sant'Andrea	Vercelli, Italy	Collabolator	The Italian SITIP-SIP Pediatric SARS-CoV-2 Infection Study Group
Alessandro	Vigo		MD	Pediatria Ospedale Michele e Pietro Ferrero	Verduno, Italy	Collabolator	The Italian SITIP-SIP Pediatric SARS-CoV-2 Infection Study Group
Stefania	Bezzio		MD	SC Pediatria, Ospedale Santa Croce	Moncalieri, Italy	Collabolator	The Italian SITIP-SIP Pediatric SARS-CoV-2 Infection Study Group
Pasquale	Comberiati		MD	AOU Pisana	Pisa, Italy	Collabolator	The Italian SITIP-SIP Pediatric SARS-CoV-2 Infection Study Group
Giovanna	Battezzati		MD	Primary Care Pediatrician		Collabolator	The Italian SITIP-SIP Pediatric SARS-CoV-2 Infection Study Group

*Indicates required information. Only first name, last name, and suffix will appear in PubMed.

***First Name and Middle Initial(s)**	***Last Name**	***Suffix (eg, Jr, III)**	Academic Degrees	Institution	Location (city, state/province, country)	Role or Contribution, eg, chair, principal investigator	Group (if more than 1 Group listed in the byline) and/or Subgroup (eg, Steering Committee)
Paola	Verna		MD	Primary Care Pediatrician		Collabolator	The Italian SITIP-SIP Pediatric SARS-CoV-2 Infection Study Group
Gianluca	Tornese		MD	Clinica Pediatrica, IRCCS Materno-Infantile “Burlo Garofolo”	Trieste, Italy	Collabolator	The Italian SITIP-SIP Pediatric SARS-CoV-2 Infection Study Group
Francesca	Poma		MD	AO Cardinal Massaia	Asti, Italy	Collabolator	The Italian SITIP-SIP Pediatric SARS-CoV-2 Infection Study Group
Luca	Bertacca		MD	Ospedale Misericordia	Grosseto, Italy	Collabolator	The Italian SITIP-SIP Pediatric SARS-CoV-2 Infection Study Group
Giorgio	Zavarise		MD	IRCCS Ospedale Sacro Cuore Don Calabria	Negrar (VE), Italy	Collabolator	The Italian SITIP-SIP Pediatric SARS-CoV-2 Infection Study Group
Luigi	Marchione		MD	Primary Care Pediatrician		Collabolator	The Italian SITIP-SIP Pediatric SARS-CoV-2 Infection Study Group
Paola	Gallia		MD	Primary Care Pediatrician		Collabolator	The Italian SITIP-SIP Pediatric SARS-CoV-2 Infection Study Group
Paola	Di Filippo		MD	Ospedale Clinicizzato di Chieti, Chieti, Italy	Chieti, Italy	Collabolator	The Italian SITIP-SIP Pediatric SARS-CoV-2 Infection Study Group
Chiara	Centenari		MD	USL Toscana Nordovest	Versilia, Italy	Collabolator	The Italian SITIP-SIP Pediatric SARS-CoV-2 Infection Study Group
Alessandra	Iacono		MD	Department of Pediatrics, Santa Maria delle Croci Hospital	Ravenna, Italy	Collabolator	The Italian SITIP-SIP Pediatric SARS-CoV-2 Infection Study Group

Supplemental Online Content: Nonauthor Collaborators

*Indicates required information. Only first name, last name, and suffix will appear in PubMed.

*First Name and Middle Initial(s)	*Last Name	*Suffix (eg, Jr, III)	Academic Degrees	Institution	Location (city, state/province, country)	Role or Contribution, eg, chair, principal investigator	Group (if more than 1 Group listed in the byline) and/or Subgroup (eg, Steering Committee)
Alessandro	Plebani		MD	Department of Experimental and Clinical Sciences, Pediatric Clinic, University of Brescia	Brescia, Italy	Collabolator	The Italian SITIP-SIP Pediatric SARS-CoV-2 Infection Study Group
Marta	Ferretti		MD	Infectious Diseases Unit, IRCCS Istituto Giannina Gaslini	Genoa, Italy	Collabolator	The Italian SITIP-SIP Pediatric SARS-CoV-2 Infection Study Group
Sara	Rizzi		MD	UOC Pediatria, Ospedale degli Infermi di Rimini	Rimini, Italy	Collabolator	The Italian SITIP-SIP Pediatric SARS-CoV-2 Infection Study Group
Sara	Rossin		MD	Division of Pediatric Infectious Diseases, Department of Women's and Children's Health, University Hospital of Padua	Padua, Italy	Collabolator	The Italian SITIP-SIP Pediatric SARS-CoV-2 Infection Study Group
Alessandro	De Fanti		MD	SC Pediatria, IRCCS AO Arcispedale Santa Maria Nuova	Reggio Emilia, Italy	Collabolator	The Italian SITIP-SIP Pediatric SARS-CoV-2 Infection Study Group
Costantino	De Giacomo		MD	Paediatrics Division, ASST Grande Ospedale Metropolitano Niguarda	Milan, Italy	Collabolator	The Italian SITIP-SIP Pediatric SARS-CoV-2 Infection Study Group
Graziano	Barera		MD	IRCCS San Raffaele Hospital	Milan, Italy	Collabolator	The Italian SITIP-SIP Pediatric SARS-CoV-2 Infection Study Group
Roberta	Pajno		MD	IRCCS San Raffaele Hospital	Milan, Italy	Collabolator	The Italian SITIP-SIP Pediatric SARS-CoV-2 Infection Study Group
Stefano	Martelossi		MD	Ospedale Cà Foncello	Treviso, Italy	Collabolator	The Italian SITIP-SIP Pediatric SARS-CoV-2 Infection Study Group

*Indicates required information. Only first name, last name, and suffix will appear in PubMed.

***First Name and Middle Initial(s)**	***Last Name**	***Suffix (eg, Jr, III)**	Academic Degrees	Institution	Location (city, state/province, country)	Role or Contribution, eg, chair, principal investigator	Group (if more than 1 Group listed in the byline) and/or Subgroup (eg, Steering Committee)
Alessia	Rocchi		MD	Fondazione IRCCS Cà Granda Ospedale Maggiore Policlinico	Milan, Italy	Collabolator	The Italian SITIP-SIP Pediatric SARS-CoV-2 Infection Study Group
Rosa	Francavilla		MD	Policlinico S.Orsola-Malpighi	Bologna, Italy	Collabolator	The Italian SITIP-SIP Pediatric SARS-CoV-2 Infection Study Group
Paola	Sogno Valin		MD	Ospedale S. Maria della Scaletta	Imola, Italy	Collabolator	The Italian SITIP-SIP Pediatric SARS-CoV-2 Infection Study Group
Piero	Valentini		MD	IRCCS Fondazione Policlinico Universitario Agostino Gemelli	Roma, Italy	Collabolator	The Italian SITIP-SIP Pediatric SARS-CoV-2 Infection Study Group
Anna Maria	Magistà		MD	Department of Pediatrics, Santa Maria delle Croci Hospital	Ravenna, Italy	Collabolator	The Italian SITIP-SIP Pediatric SARS-CoV-2 Infection Study Group
Carolina	Saffioti		MD	Infectious Diseases Unit, IRCCS Istituto Giannina Gaslini	Genoa, Italy	Collabolator	The Italian SITIP-SIP Pediatric SARS-CoV-2 Infection Study Group
Maria Cristina	Greco		MD	Primary Care Pediatrician		Collabolator	The Italian SITIP-SIP Pediatric SARS-CoV-2 Infection Study Group
Paola	Costenaro		MD	Primary Care Pediatrician		Collabolator	The Italian SITIP-SIP Pediatric SARS-CoV-2 Infection Study Group
Maria	Di Pietro		MD	Primary Care Pediatrician		Collabolator	The Italian SITIP-SIP Pediatric SARS-CoV-2 Infection Study Group
Susanna	Esposito		MD	Pietro Barilla Children's Hospital and Department of Medicine and Surgery, University of Parma	Parma, Italy	Collabolator	The Italian SITIP-SIP Pediatric SARS-CoV-2 Infection Study Group

*Indicates required information. Only first name, last name, and suffix will appear in PubMed.

***First Name and Middle Initial(s)**	***Last Name**	***Suffix (eg, Jr, III)**	Academic Degrees	Institution	Location (city, state/province, country)	Role or Contribution, eg, chair, principal investigator	Group (if more than 1 Group listed in the byline) and/or Subgroup (eg, Steering Committee)
Giovanni	Pieri		MD	Pediatric and Pediatric Emergency Unit, The Children Hospital, AO SS Antonio e Biagio e C. Arrigo	Alessandria, Italy	Collabolator	The Italian SITIP-SIP Pediatric SARS-CoV-2 Infection Study Group
Maria Valentina	Spartà		MD	UOC Pediatria ASST di Lodi	Lodi, Italy	Collabolator	The Italian SITIP-SIP Pediatric SARS-CoV-2 Infection Study Group
Valentina	Maffini		MD	Emergency and General Pediatric Unit, Pietro Barilla Children's Hospital	Parma, Italy	Collabolator	The Italian SITIP-SIP Pediatric SARS-CoV-2 Infection Study Group
Francesco	Licciardi		MD	Regina Margherita Children's Hospital, University of Turin	Turin, Italy	Collabolator	The Italian SITIP-SIP Pediatric SARS-CoV-2 Infection Study Group
Carlo	Scolfaro		MD	Pediatric Infectious Diseases Unit, Regina Margherita Children's Hospital, University of Turin, Turin, Italy	Turin, Italy	Collabolator	The Italian SITIP-SIP Pediatric SARS-CoV-2 Infection Study Group
Giacomo	Stera		MD	Pediatric Emergency Unit, IRCCS Azienda Ospedaliero-Universitaria di Bologna,	Bologna, Italy	Collabolator	The Italian SITIP-SIP Pediatric SARS-CoV-2 Infection Study Group
Agostina	Marolda		MD	ASST Ovest Milanese, Ospedale Fornaroli	Magenta, Italy	Collabolator	The Italian SITIP-SIP Pediatric SARS-CoV-2 Infection Study Group
Lorenzo	Fiorica		MD	Division of Pediatrics and Neonatology, Department of Maternal, Neonatal, and Infant Health, Ospedale degli Infermi	Biella, Italy	Collabolator	The Italian SITIP-SIP Pediatric SARS-CoV-2 Infection Study Group
Caterina	Sabatini		MD	UOC Pediatria ASST di Lodi	Lodi, Italy	Collabolator	The Italian SITIP-SIP Pediatric SARS-CoV-2 Infection Study Group

Supplemental Online Content: Nonauthor Collaborators

*Indicates required information. Only first name, last name, and suffix will appear in PubMed.

***First Name and Middle Initial(s)**	***Last Name**	***Suffix (eg, Jr, III)**	Academic Degrees	Institution	Location (city, state/province, country)	Role or Contribution, eg, chair, principal investigator	Group (if more than 1 Group listed in the byline) and/or Subgroup (eg, Steering Committee)
Maria Rita	Genovese		MD	Clinica Pediatrica, IRCCS Materno-Infantile “Burlo Garofolo”	Trieste, Italy	Collabolator	The Italian SITIP-SIP Pediatric SARS-CoV-2 Infection Study Group
Cristina	Ferrari		MD	Pediatric and Pediatric Emergency Unit, The Children Hospital, AO SS Antonio e Biagio e C. Arrigo	Alessandria, Italy	Collabolator	The Italian SITIP-SIP Pediatric SARS-CoV-2 Infection Study Group
Désirée	Caselli		MD	Ospedale Pediatrico Giovanni XXIII	Bari, Italy	Collabolator	The Italian SITIP-SIP Pediatric SARS-CoV-2 Infection Study Group
Francesco	Chiarelli		MD	Dipartimento di medicina e scienze dell’invecchiamento, Università degli Studi “G. D’Annunzio” Chieti e Pescara	Chieti, Italy	Collabolator	The Italian SITIP-SIP Pediatric SARS-CoV-2 Infection Study Group
Franco	Di Lollo		MD	ASL Teramo, UOC Pediatria e Neonatologia	Teramo, Italy	Collabolator	The Italian SITIP-SIP Pediatric SARS-CoV-2 Infection Study Group
Cesare	Ghitti		MD	ASST Bergamo Est	Seriate, Italy	Collabolator	The Italian SITIP-SIP Pediatric SARS-CoV-2 Infection Study Group
Caterina	Marabotto		MD	Universitarian Hospital Department, Ospedale Pediatrico Bambino Gesù, IRCCS	Roma, Italy	Collabolator	The Italian SITIP-SIP Pediatric SARS-CoV-2 Infection Study Group
Raffaele	Papa		MD	Universitarian Hospital Department, Ospedale Pediatrico Bambino Gesù, IRCCS	Roma, Italy	Collabolator	The Italian SITIP-SIP Pediatric SARS-CoV-2 Infection Study Group

Supplemental Online Content: Nonauthor Collaborators

*Indicates required information. Only first name, last name, and suffix will appear in PubMed.

*First Name and Middle Initial(s)	*Last Name	*Suffix (eg, Jr, III)	Academic Degrees	Institution	Location (city, state/province, country)	Role or Contribution, eg, chair, principal investigator	Group (if more than 1 Group listed in the byline) and/or Subgroup (eg, Steering Committee)
Laura	Cursi		MD	Universitarian Hospital Department, Ospedale Pediatrico Bambino Gesù, IRCCS	Roma, Italy	Collabolator	The Italian SITIP-SIP Pediatric SARS-CoV-2 Infection Study Group
Sandra	Trapani		MD	Department of Health Sciences, University of Florence	Florence, Italy	Collabolator	The Italian SITIP-SIP Pediatric SARS-CoV-2 Infection Study Group
Anna Elisabetta	Bussolini		MD	SC Pediatria, ASST Settelaghi	Varese, Italy	Collabolator	The Italian SITIP-SIP Pediatric SARS-CoV-2 Infection Study Group
